# A rare case of teratoma in the right atrium: A case report study

**DOI:** 10.1016/j.ijscr.2024.110448

**Published:** 2024-10-12

**Authors:** Ahmad Alkheder, Ibrahim Fathallah, Abd Alrhman Alajrd, Ahmed Al-Talep, Zeina Alsodi, Saleh Takkem

**Affiliations:** a Department of Otorhinolaryngology, Al Mouwasat University Hospital, Faculty of Medicine, Damascus University, Damascus, Syria; b Faculty of Medicine, Syrian Private University, Damascus, Syria; c Faculty of Medicine, Damascus University, Damascus, Syria; d Faculty of Medicine, Al-Baath University, Homs, Syria; e Department of Cardiology, Alwatani Hospital, Hama University, Hama, Syria

**Keywords:** Teratoma, Heart, Atrium, Murmur, Rare case

## Abstract

**Introduction:**

Teratomas are neoplasms originating from embryonic tissues, characterized by a diverse composition of cells from all three germ layers in varying ratios. Rarely reported in the heart, we present here a rare case of a teratoma in the right atrium of a newborn.

**Case presentation:**

A 20-h-old newborn was referred for a mild heart murmur. An echocardiogram revealed a 6 × 9 mm mass in the right atrium, attached to the interatrial septum. The mass caused mild tricuspid regurgitation without any significant pressure gradient. Surgery to remove the mass was successful. Pathological examination confirmed the mass as a teratoma. The child was discharged in excellent health and has had normal follow-up exams.

**Discussion:**

Mature teratomas, a common type of germ cell tumor, are rare in the mediastinum, accounting for 10–15 % of mediastinal masses. These tumors arise from displaced primordial germ cells, which may become malignant. They can metastasize to the heart via various routes, causing symptoms like cough, dyspnea, and chest pain due to compression of mediastinal structures. Severe cases may lead to superior vena cava syndrome or hemoptysis. Tumor location influences clinical presentation, with some posing life-threatening risks.

**Conclusion:**

Early diagnosis and intervention were crucial in preventing severe complications. This case emphasizes the importance of vigilant clinical evaluation and timely surgical management for infants presenting with cardiac symptoms.

## Highlights

• A rare case of a right atrial teratoma in a newborn, presenting with a mild heart murmur.
• Teratomas, though more common in other regions, are extremely rare in the heart.
• This case emphasizes the importance of early detection and timely surgical intervention to prevent serious outcomes.

## 1 Introduction

Teratomas are neoplasms originating from embryonic tissues, characterized by a diverse composition of cells from all three germ layers in varying ratios. The term ‘teratoma’ is derived from the Greek words ‘terato,’ meaning ‘monster,’ and ‘onkoma,’ meaning ‘tumor’ or ‘swelling.’ Although the majority of teratomas are benign, there is a potential for malignancy [1,2]. These tumors are most frequently found in the sacrococcygeal region (40 %), ovaries (25 %), testes (12 %), and brain (5 %). Other locations, such as the neck and mediastinum, account for the remaining 18 % of cases. Teratomas constitute a minor fraction of primary cardiac tumors, representing only 7 % of all cardiac neoplasms [3]. Cardiac teratomas predominantly affect the pericardium, with a smaller proportion occurring within the myocardium. Primary cardiac tumors in neonates are exceedingly rare, with an incidence of approximately 1 in 100,000 live births [4]. Newborns or fetuses with teratomas may exhibit symptoms due to the tumor's mass effect and the accumulation of pericardial fluid. Common symptoms include cyanosis, congestive heart failure, and respiratory distress [3,4]. It is crucial for pediatricians to prioritize early diagnosis and timely intervention for teratomas to prevent the condition from becoming life-threatening. In this report, we present a rare case of a 20-h-old infant diagnosed with a teratoma located in the right atrium.

This work is also reported in line with SCARE criteria which helped to improve the transparency and quality of this case report [5].

## 2 Case presentation

**Fig. 1:**
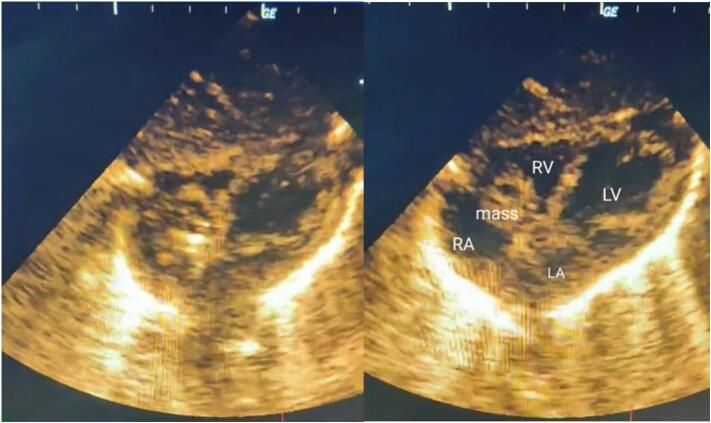
Echocardiogram shows a mass in the right atrium and prolapse of the mass across the tricuspid valve. RV: right ventricle, LV: left ventricle, RA: right atrium, LA: left atrium.

**Fig. 2:**
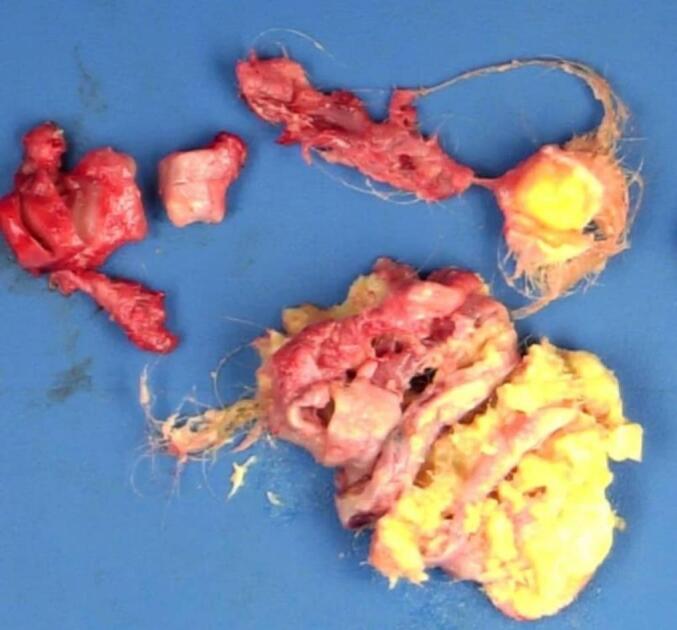
The excised tumor mass, measuring approximately 10 × 12 mm, shows variably differentiated tissues.

**Fig. 3:**
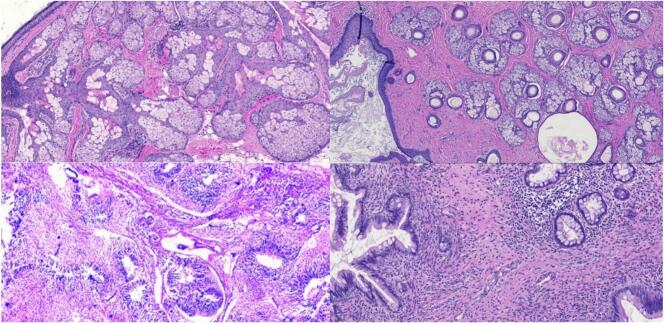
Histopathologic examination, showed the tumor cells made up of all three germ layers: ectoderm, mesoderm, and endoderm. The tissue shows well-differentiated cartilaginous, squamous epithelial, glandular, and intestinal columnar epithelial.

A newborn, only 20 h old, was delivered vaginally to a 35-year-old mother and subsequently referred to the cardiac clinic for assessment of a mild, grade 2/6 systolic murmur at the tricuspid valve. The infant exhibited no symptoms and had normal APGAR scores. Vital signs were stable, and initial lab results were within normal ranges, except for a slightly elevated CRP level of 7.5 mg/L. An echocardiogram revealed normal organ positioning, with the superior and inferior vena cavae draining into the right atrium and proper atrioventricular and ventriculoarterial alignments. The aortic root appeared normal, though a hyperdynamic abdominal aorta was observed. Both ventricles showed good function and normal motion, and the mitral, aortic, and pulmonary valves were normal. A 6 × 9 mm heterogeneous mass was detected in the right atrium, attached to the interatrial septum (Fig. 1). This mass caused mild tricuspid regurgitation without a significant pressure gradient (mean gradient across the tricuspid valve: 1.5 mmHg) and was mobile within the right atrium (Video 1). The interatrial septum, interventricular septum, and left aortic arch were intact. The child was referred for cardiac surgery. Due to the clear echocardiogram, no further investigations were necessary. The infant underwent surgery at the age of 10 days to remove the mass, which was occupying the right atrium (Fig. 2). The procedure involved a median sternotomy to access and open the right atrium for tumor removal. A routine pacing wire was placed post-surgery and removed after three days. No medications or additional support were required post-operatively. Pathological examination identified the mass as a teratoma attached to the interatrial septum (Fig. 3). The child was discharged in excellent health three days after surgery. Follow-up echocardiograms at six and twelve months post-surgery were normal. The child is currently doing well and continues to have regular check-ups at our clinic.

## 3 Discussion

Mature teratomas represent a subset of germ cell tumors and are recognized as the most frequent histological variant within this category. While primary germ cell tumors account for 10–15 % of all mediastinal masses, their occurrence in the mediastinum remains relatively uncommon [6]. The incidence of mediastinal tumors can be ranked in the following order: striated muscle tumors, fibrous tumors, teratomas, and lipomas [7]. The development of extragonadal germ cell tumors is often linked to abnormal or incomplete migration of primordial germ cells from the yolk sac to the gonads. These displaced germ cells may undergo malignant transformation, particularly along the urogenital ridge [8]. Cardiac metastasis from these tumors can occur through various routes, including direct invasion, lymphatic spread, hematogenous dissemination, or intracavitary extension via the superior vena cava or pulmonary veins [8]. Germ cell tumors in the mediastinum can present significant clinical challenges, often due to their tendency for aggressive growth, which can compress nearby mediastinal structures [9]. As a result, patients may experience symptoms such as persistent cough, difficulty breathing, palpitations, chest or back pain, and other signs related to the pressure exerted by the tumor on surrounding tissues [10]. The compression of major blood vessels can impair circulation. For instance, compression of the superior vena cava may lead to superior vena cava syndrome, a critical medical condition. Furthermore, this vascular compression can result in vessel blockage, tissue ischemia, and necrosis. In some cases, hemoptysis may develop as a result of bronchial wall necrosis caused by tumor invasion [9]. Teratomas, known for their rapid growth, can have severe consequences due to mechanical compression of adjacent structures [11]. The location of the tumor is crucial in determining its clinical presentation. While some tumors may be asymptomatic, others—though rare—can pose life-threatening risks. Symptoms may arise through mechanisms such as invasion of the heart or lungs, or even embolism [12]. Tumors can originate from various cardiac structures, such as the endocardium or pericardium, as seen with lipomas. Fibromas, on the other hand, are typically located within the ventricular myocardium, while rhabdomyomas often arise in the ventricular septum, particularly the left ventricle. In the mediastinum, these tumors frequently appear in the anterior region, often involving the great vessels' base [11]. In our reported case, a mobile teratoma was identified within the right atrium, attached to the interatrial septum. This mass contributed to mild tricuspid regurgitation and had a hyperdynamic effect on the abdominal aorta. Surgical excision of the right atrial teratoma was successfully performed.

## 4 Conclusion

In this case report, we presented a rare instance of a 20-h-old infant diagnosed with a benign teratoma located in the right atrium. The infant exhibited mild symptoms and was successfully treated with surgical excision of the tumor. While cardiac teratomas are uncommon, this case highlights the importance of early diagnosis and prompt intervention to prevent severe complications. The successful outcome of this case underscores the significance of vigilant clinical evaluation and timely surgical management for infants presenting with cardiac murmurs or other concerning symptoms.

The following is the supplementary data related to this article.

Supplementary Video 1 Supplementary Video 1

## Consent of patient

Written informed consent was obtained from the patient's parents/legal guardian for publication and any accompanying images. A copy of the written consent is available for review by the Editor-in-Chief of this journal on request.

## Provenance and peer review

Not commissioned, externally peer-reviewed.

## Ethical approval

Ethics clearance was not necessary since the University waives ethics approval for publication of case reports involving no patients' images, and the case report is not containing any personal information. The ethical approval is obligatory for research that involve human or animal experiments.

## Guarantor

The corresponding author takes the full responsibility of the work.

## Research registration number

This case report is not a first time of reporting, new device or surgical technique. So I would not need a Research Registry Unique identifying number (UIN).

## Funding

N/A. We received no funding in any form.

## Author contribution

**Ahmad Alkheder and Zeina Alsodi:** Validation, Writing – review & editing, Visualization, Methodology, Software, Writing – original draft, Formal analysis. **Ibrahim Fathallah and Abd Alrhman Alajrd and Ahmed Al-Talep**: Validation, Writing – review & editing, Visualization, Methodology, Software, Writing – original draft, Formal analysis. **Saleh Takkem**: Supervision, Writing – review & editing, Project administration.

## Conflict of interest statement

The Authors disclose no conflicts.

## Data Availability

All data are available from the corresponding author on reasonable request. The case has not been presented at a conference or regional meeting.
