# Corrigendum to “A rare case of teratoma in the right atrium: A case report study” [Int. J. Surg. Case Rep. 124 (2024) 110448]

**DOI:** 10.1016/j.ijscr.2024.110565

**Published:** 2024-11-12

**Authors:** Ahmad Alkheder, Ibrahim Fathallah, Abd Alrhman Alajrd, Ahmed Al-Talep, Zeina Alsodi, Saleh Takkem

**Affiliations:** a Department of Otorhinolaryngology, Al Mouwasat University Hospital, Faculty of Medicine, Damascus University, Damascus, Syria; b Faculty of Medicine, Syrian Private University, Damascus, Syria; c Faculty of Medicine, Damascus University, Damascus, Syria; d Faculty of Medicine, Al-Baath University, Homs, Syria; e Department of Cardiology, Alwatani Hospital, Hama University, Hama, Syria

The authors regret uploading an inappropriate video. The uploaded portion of the video does not show the tumor. We have attached the correct video that clearly shows the tumor during the ultrasound procedure.

The authors regret uploading an inappropriate video. The uploaded portion of the video does not show the tumor. We have attached the correct video that clearly shows the tumor during the ultrasound procedure.

The authors would like to apologise for any inconvenience caused.

The following is the supplementary data related to this article.

Supplementary video Supplementary video

Supplementary data to this article can be found online at https://doi.org/10.1016/j.ijscr.2024.110565.

